# Surprising Presentation of Intra-abdominal Tuberculosis: A Case Report

**DOI:** 10.7759/cureus.80214

**Published:** 2025-03-07

**Authors:** Fathi Elgeyoushy, Taif H Alomar, Mohammad M Alghabban, Rema I Alshagary, Abdulmajeed M Alamri

**Affiliations:** 1 General Surgery, King Fahad General Hospital, Medina, SAU; 2 General Surgery, King Faisal Specialist Hospital and Research Centre, Medina, SAU

**Keywords:** abdomen pain, gallbladder diseases and gallstones, intestinal tuberculosis, open cholecystectom, tb, tuberculosis

## Abstract

Tuberculosis (TB) is a chronic infectious disease caused by *Mycobacterium tuberculosis*, posing a significant global health challenge, especially in low- and middle-income countries. While it primarily affects the lungs, it can also involve other organs, leading to varied clinical manifestations. We present the case of a 49-year-old male who experienced moderate right upper quadrant pain, nausea, and tenderness for one month. Initially diagnosed with acute cholecystitis based on clinical evaluation and imaging, intraoperative findings revealed disseminated peritoneal nodules and ascitic fluid, raising suspicion of abdominal TB. A retrograde cholecystectomy and right hemicolectomy were performed, but both acid-fast bacilli (AFB) staining and polymerase chain reaction (PCR) tests returned negative results. However, histopathological examination of the gallbladder ultimately confirmed TB, prompting the initiation of treatment. This case highlights the diagnostic challenges of abdominal TB, where clinical, radiological, and molecular tests may fail to provide conclusive results. Histopathology plays a critical role in diagnosis, and effective management often requires a combination of surgical intervention and prolonged anti-TB therapy. Early recognition of abdominal TB, particularly in atypical presentations, along with a multidisciplinary approach that includes both surgical and medical management, is vital for achieving successful outcomes. Prioritizing histopathological examination is essential when other diagnostic tests yield inconclusive results.

## Introduction

In Saudi Arabia, tuberculosis (TB) surveillance and reporting began in 1970, when the incidence rate was 1,298.5 cases per 100,000 people. This rate gradually decreased, dropping to 135 cases per 100,000 in 1980 and further to 12 cases per 100,000 by 1997. However, from 2000 to 2009, the incidence rate stabilized at around 15-16 cases per 100,000, even as the total number of new cases increased from 3,284 to 3,964 [1]. Among the various forms of extrapulmonary TB, abdominal TB ranks as the sixth most common, following lymphatic, genitourinary, bone and joint, miliary, and meningeal TB [2]. Abdominal TB accounts for up to 12% of all extrapulmonary TB cases and remains particularly prevalent in developing nations such as India, Saudi Arabia, and South Africa [2]. Furthermore, the incidence of TB is rising in developed countries, largely due to the increasing number of immunocompromised individuals, primarily as a result of the AIDS pandemic, along with growing immigrant populations, worsening social conditions, and reductions in health services [3]. Abdominal TB can manifest in various anatomical locations, including the peritoneum, esophagus, intestines, lymph nodes, hepatobiliary system, stomach, perianal area, and pancreas [4].

## Case presentation

A 49-year-old male patient, with no known medical history, previous surgical procedures, or allergies, presented to our outpatient clinic with a complaint of moderate, colicky right upper quadrant pain that had persisted for one month. The pain developed gradually, reaching a severity of 6 out of 10, and was aggravated by the intake of fatty meals. It was localized without radiation, and no alleviating factors were identified. The patient also reported associated nausea with increased pain severity, but there were no episodes of vomiting, changes in bowel habits, urinary symptoms, weight loss, or fever. A review of other systems was unremarkable. Family history was also unremarkable. It is important to note that the patient did not exhibit any identifiable risk factors, as he had no history of immunocompromising conditions such as human immunodeficiency virus (HIV) infection. Additionally, he had not been exposed to crowded living conditions or environments known for high TB prevalence.

**Figure 1:**
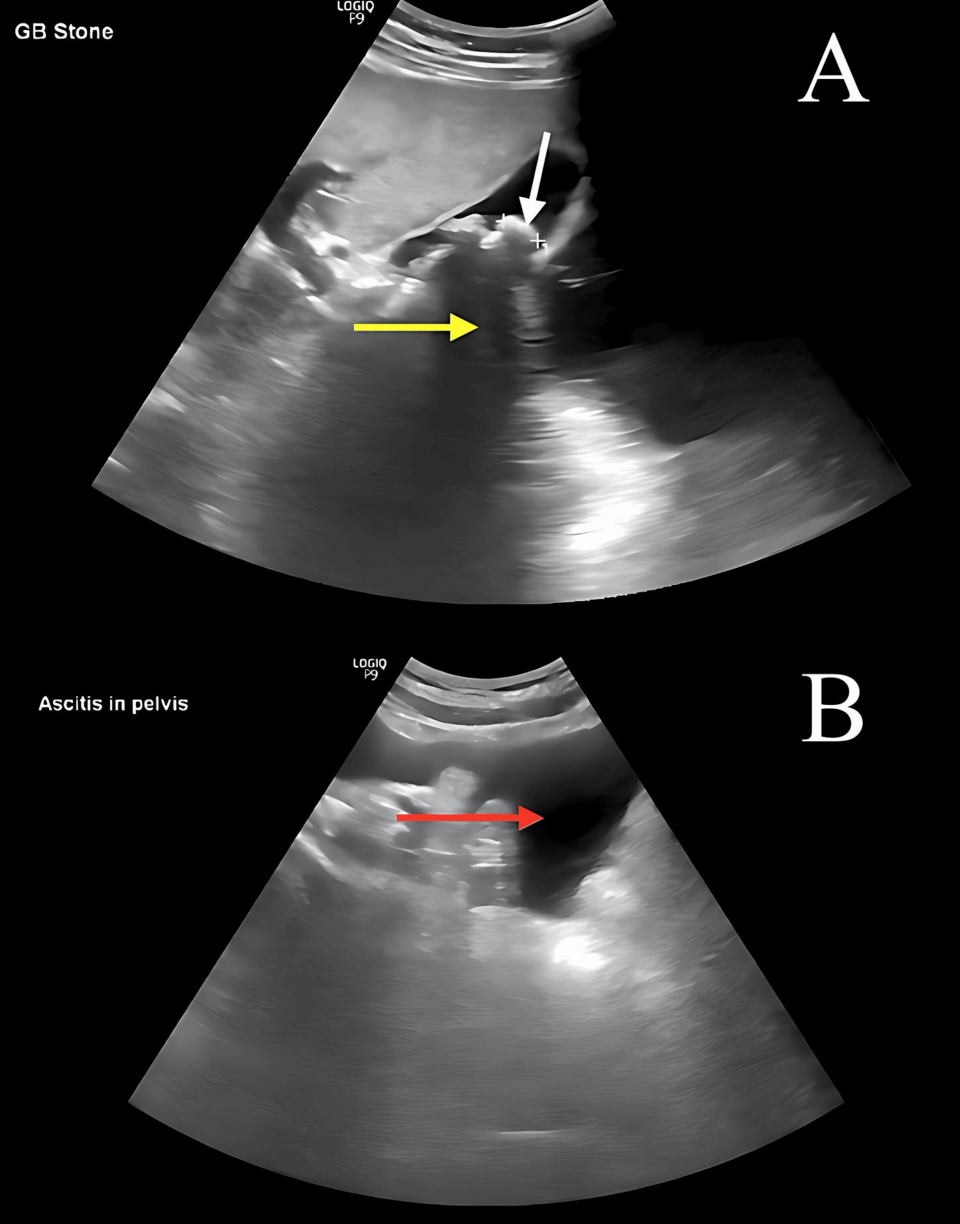
Abdomen and pelvis ultrasound Yellow arrow: posterior acoustic shadow.  White arrow: gallbladder stones. Red arrow: ascites In the pelvis.

The general physical examination indicated normal vital signs and a body mass index (BMI) of 26.6, with a weight of 75 kg and height of 168 cm. Local examination of the abdomen revealed tenderness in the right upper quadrant and a positive Murphy sign. There were no skin changes, hernial orifices were intact, and both superficial and deep abdominal palpation did not reveal any significant findings. Abdominal auscultation showed normal bowel sounds, and the examination of other systems was unremarkable. An abdominal ultrasound confirmed the diagnosis of acute cholecystitis (Figure 1A) and showed ascites (Figure 1B). The patient subsequently underwent a preoperative assessment, which included a complete blood count (CBC), liver function tests (LFTs), kidney function tests (KFTs), and coagulation profile (Table 1). Both the general surgery and anesthesiology teams assessed the patient and declared him fit for surgical management within two weeks of presentation.

**Table 1:** Laboratory findings

Investigation	Result	Normal Range
White blood cells	7.9 × 10^9^/L	4–10 × 10^9^/L
Red blood cells	5.32 × 10^12^/L	4.5–5.5 × 10^12^/L
Haemoglobin	14.2 g/dL	13–17 g/dL
Platelets	361 × 10^9^/L	150–410 × 10^9^/L
Urea	3.3 mmol/L	2.5–6.43 mmol/L
Creatinine	55 µmol/L	53–106 µmol/L
Sodium	136 mmol/L	136–145 mmol/L
Potassium	4.3 mmol/L	3.5–5.3 mmol/L
Aspartate aminotransferase	37 U/L	10–50 U/L
Alanine transaminase	35 U/L	0–41 U/L
Total bilirubin	13 µmol/L	0–20.5 µmol/L
Conjugated bilirubin	3.5 µmol/L	0–5 µmol/L

**Figure 2:**
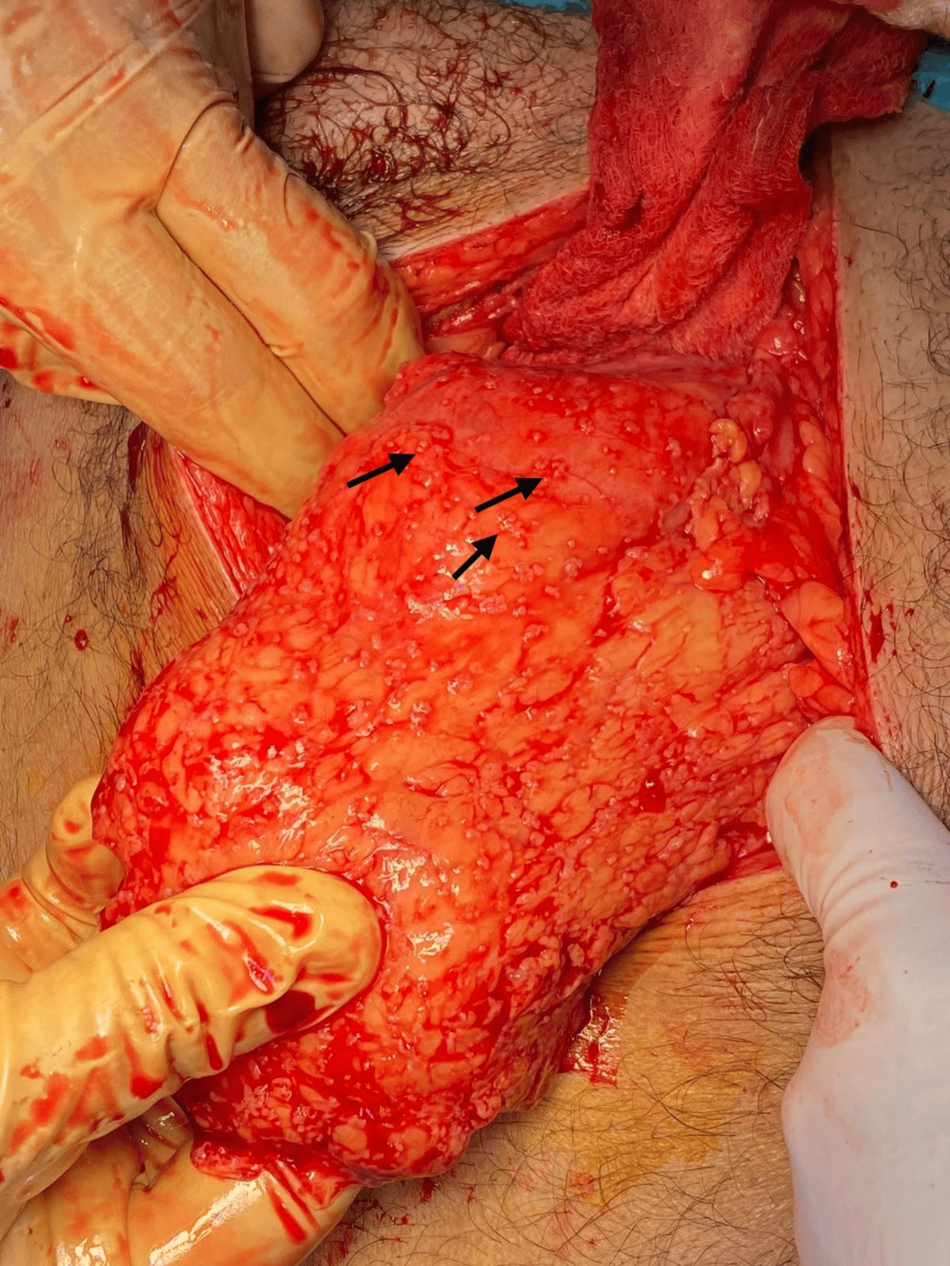
Omentum nodules Disseminated nodules are observed all over the omentum.

**Figure 3:**
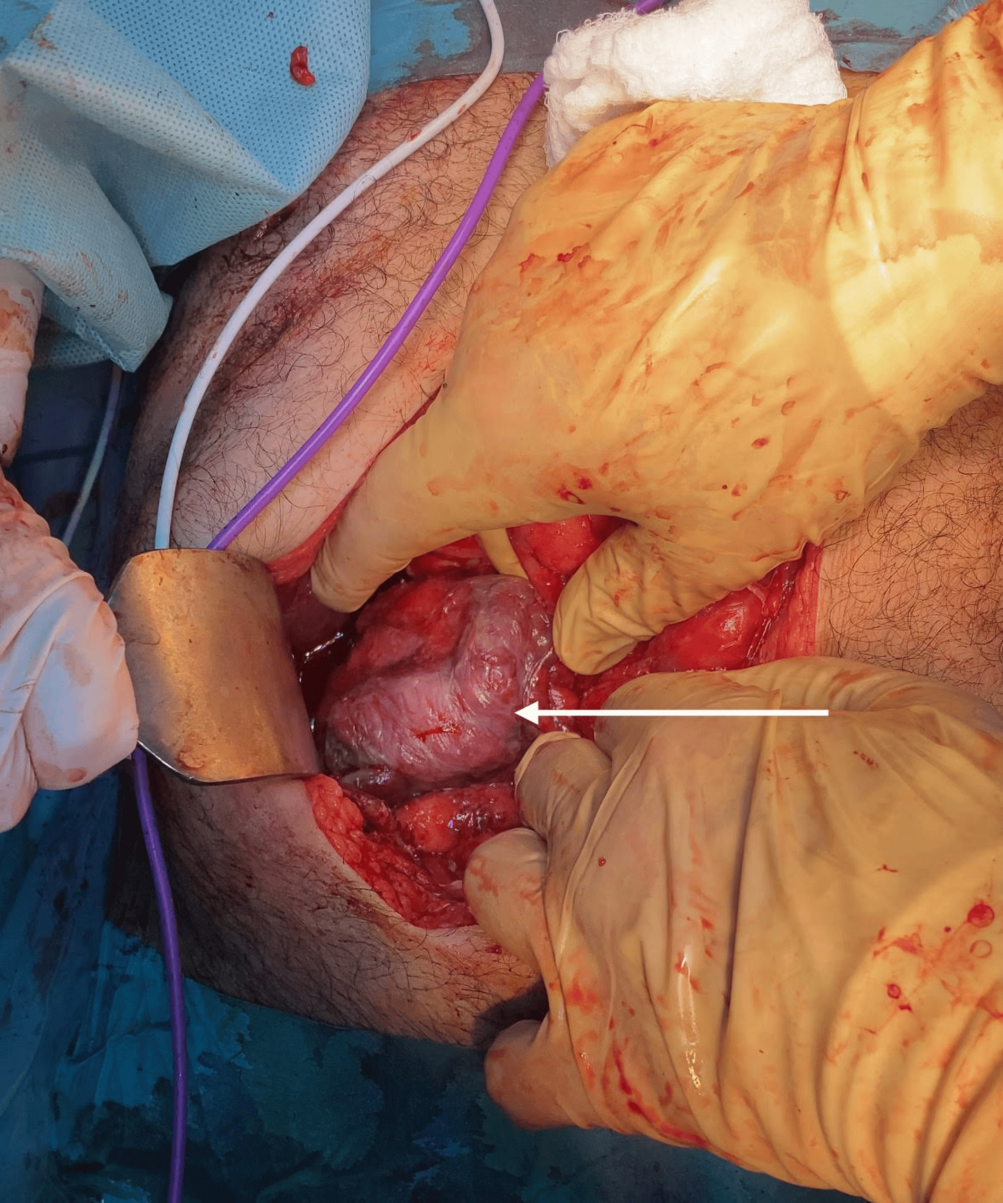
Cecum A dusky-colored cecum is observed.

On the day of surgery, the patient was stable and placed in a supine position, and general anesthesia was administered. The abdomen was insufflated using a Veress needle, and four trocars were introduced. Upon visual inspection of the operative field, multiple adhesions were observed near the gallbladder, along with disseminated small nodules across the anterior abdominal wall and visceral peritoneum, including the omentum, colon, small bowel, liver, and stomach (Figure 2), with a moderate amount of hemorrhagic ascitic fluid. Due to these findings, a decision was made to convert to an open laparotomy. A midline incision was made, and the peritoneum was opened. A retrograde cholecystectomy was performed after identifying and separately ligating the cystic artery and cystic duct. During the procedure, bleeding from a small artery in the posterior peritoneum was noted and subsequently ligated. The cecum appeared dusky and unhealthy (Figure 3), although the appendix was normal, and no palpable intra-abdominal masses were found in the colon or stomach. Consequently, a right hemicolectomy with terminal ileostomy was performed. Hemostasis was achieved, and two drains were placed in the pelvis and right paracolic gutter. The laparotomy wound was closed, and a colostomy bag was applied. The patient was then transferred to the recovery room and subsequently to the general surgery ward for further evaluation and observation.

**Figure 4:**
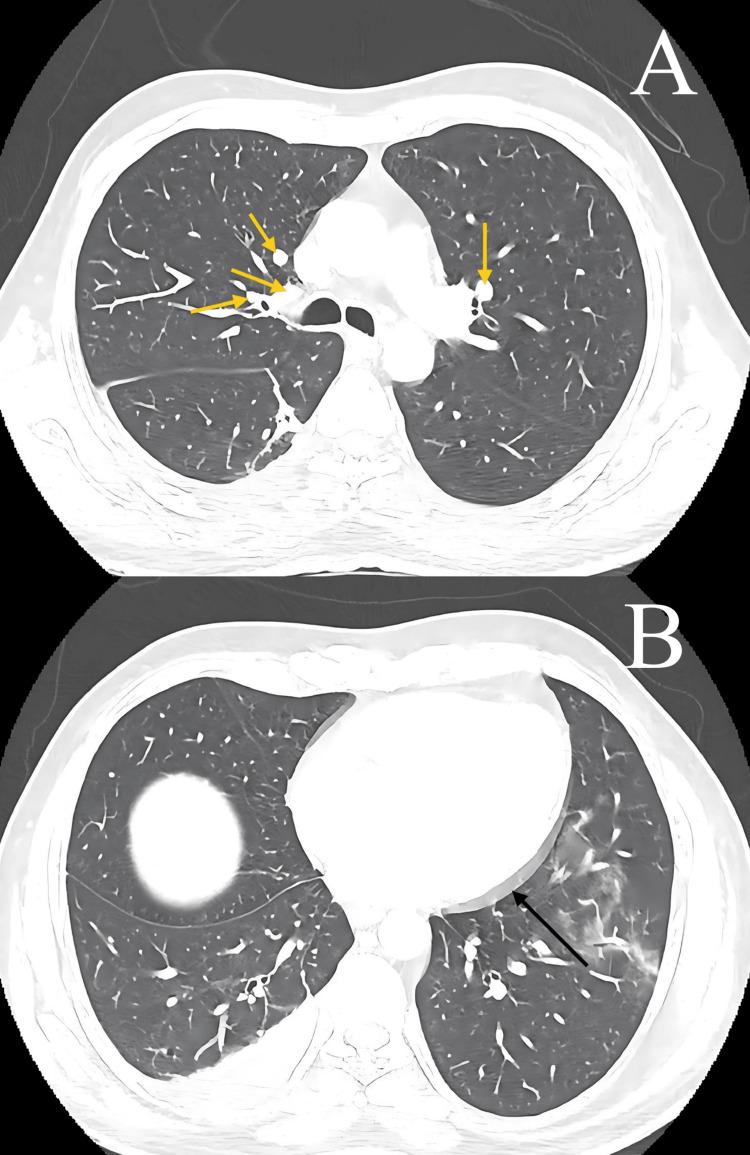
Chest computed tomography (A) Mediastinal lymphadenopathy (yellow arrows). (B) Pericardial effusion (black arrow).

Postoperatively, the patient was placed in a private room, and standard and contact precautions were implemented. He also underwent chest and abdominal CT scans to rule out masses, and an infectious panel was conducted. Tumor markers, including cancer antigen (CA) 125, CA 15-3, CA 19-9, carcinoembryonic antigen (CEA), and prostate-specific antigen (PSA) (Table 2), were tested to rule out malignancies. The results were significant for an elevated CA-125 level of 288 IU/mL. Infectious panel testing and polymerase chain reaction (PCR) testing for TB and acid-fast bacilli (AFB) were all negative. Chest CT revealed multiple enlarged mediastinal lymph nodes and pericardial effusion (Figure 4).

**Table 2:** Tumor markers

Markers	Result	Normal Range
CA 19.9	6 IU/mL	0–35
Carcinoembryonic antigen	3 ng/mL	0–3
CA 15-3	21 IU/mL	0–32
CA 125	288 IU/mL	0–35

**Figure 5:**
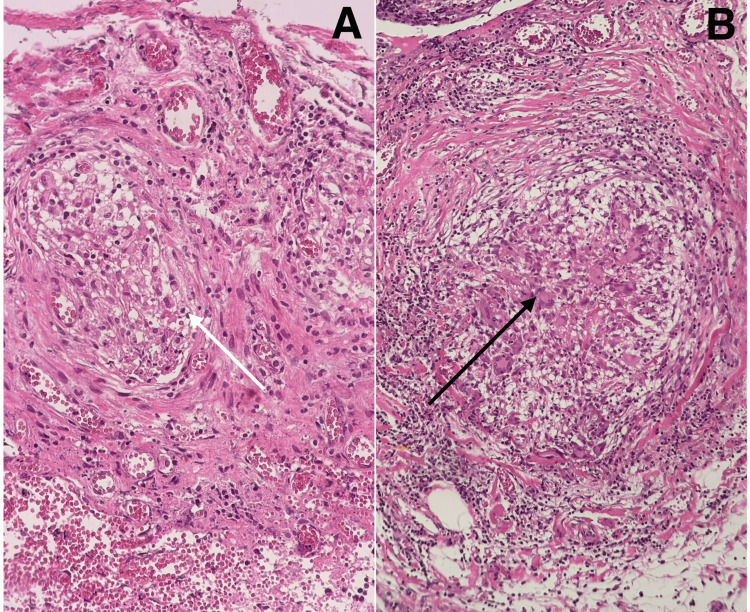
Histopathological examination (A) Necrotizing granulomatous inflammation of the colon (white arrow). (B) Chronic cholecystitis with necrotizing granulomatous inflammation in the serosa (black arrow).

The histopathological examination revealed chronic calculous cholecystitis in the gallbladder, characterized by necrotizing granulomatous inflammation in the serosa and surrounding fat. The gallbladder measured 9 × 3 cm and exhibited a hemorrhagic surface, with cut sections showing the presence of stones. Additionally, the colon resection specimen, measuring 18 cm in length, with an appendix measuring 8 × 1 cm and surrounding fat tissue measuring 14 × 5 cm, displayed necrotizing granulomatous inflammation. The surface of the colon was notably black and hemorrhagic, featuring very small multiple nodules (Figure 5).

The patient’s management involved a multidisciplinary team, including general surgery, infectious diseases, and the chest department. He was hospitalized until full recovery in a private room, with careful attention given to ensure that every healthcare provider caring for him wore personal protective equipment (PPE). The patient then continued follow-up in the outpatient clinic for anti-TB treatment. The anti-TB regimen included an initial phase of two months with isoniazid (INH), rifampin (RIF), pyrazinamide (PZA), ethambutol (EMB), and pyridoxine, followed by a continuation phase of eight months with isoniazid, rifampin, and pyridoxine. The patient reported only mild symptoms of hand arthralgia, which were managed in consultation with the rheumatology department. There were no auditory, visual, gastrointestinal, or neurological side effects. After finishing the anti-TB treatment, the patient was scheduled for stoma closure. Intraoperatively, a side-to-side anastomosis with the transverse colon was performed. The patient tolerated the procedure well and was observed in the general ward for one week until full recovery. Follow-up in the clinic was conducted at two- to three-week intervals, during which he continued doing well and did not report any abnormal symptoms.

## Discussion

TB is a disease that can involve almost any organ or system in the human body, manifesting in a wide array of radiologic patterns that may mimic other diseases, including malignancies. Among its extrapulmonary forms, abdominal TB is particularly common, with its prevalence increasing due to factors such as incomplete treatment, multidrug-resistant strains, and the rising prevalence of HIV infection [5]. Several risk factors contribute to the development of abdominal TB. These include underlying medical conditions such as cirrhosis, diabetes mellitus, HIV infection, renal insufficiency, and malignancies; the use of medications like steroids and anti-tumor necrosis factor (TNF) agents; as well as lifestyle factors like malnutrition, tobacco smoking, and alcohol consumption [6].

Abdominal TB is believed to occur through the reactivation of dormant bacilli, which may have established a primary gastrointestinal focus earlier in life. This initial focus is thought to result from the hematogenous spread of *Mycobacterium tuberculosis* from a pulmonary site, typically acquired during a primary infection in childhood. Additionally, abdominal TB may arise from swallowed bacilli that penetrate the Peyer’s patches of the intestinal mucosa, traveling through the lymphatic system via macrophages to mesenteric lymph nodes, where the bacilli remain dormant [7].

The most commonly affected site of gastrointestinal TB is the ileocecal region, followed by the jejunum and colon. TB involving the esophagus, stomach, and duodenum is rare, and gallbladder TB is considered extremely uncommon. The resistance of the gallbladder to TB is attributed to its high alkalinity and bile acid content, which inhibit the growth of tubercle bacilli [3,8]. However, conditions such as cholelithiasis and cystic duct obstruction have been identified as key factors that may predispose the gallbladder to tuberculous infection [9].

Four distinct forms of gallbladder TB have been described in the literature: (1) as part of miliary TB in both children and adults, (2) as a component of disseminated abdominal TB, (3) as isolated gallbladder TB without involvement of other body sites, and (4) as gallbladder involvement in immunocompromised states [10]. When TB affects the gastrointestinal tract, three types of lesions are typically identified: ulcerative, hypertrophic, and stricturous lesions [7].

The clinical presentation of abdominal TB can vary significantly, including acute, chronic, or acute-on-chronic forms, and it may sometimes be discovered incidentally during laparotomy performed for unrelated causes. Common symptoms include abdominal pain, weight loss, constipation, anorexia, fever, and abdominal distension [11].

Diagnosing abdominal TB generally involves a three-stage approach. The first two stages, clinical evaluation and radiological imaging, provide indirect evidence of the disease. The third stage relies on invasive methods to obtain direct confirmation. Despite the utility of these approaches, the diagnostic process remains challenging, as these methods often yield indirect evidence in practice [12].

Radiological imaging plays a crucial role in aiding diagnosis. For example, ultrasound can detect small amounts of ascites, identify septations, strands of fibrin, and reveal debris [5]. Computed tomography (CT) imaging is even more effective, identifying features such as high-density ascites, lymphadenopathy, bowel wall thickening, and irregular soft tissue densities in the omental region [2]. Although clinical and radiological findings are highly useful in assessing abdominal TB, they are not definitive diagnostic tools. Confirmatory evidence requires bacteriological and histological testing to detect the causative organism [3,13]. PCR assays, performed on biopsy specimens or ascitic fluid, have been proposed as diagnostic tools. However, their routine use is limited due to high false-negative rates [14].

When considering the differential diagnosis for abdominal TB, several conditions must be evaluated due to overlapping clinical features. Crohn's disease can mimic the granulomatous inflammation seen in TB. Infectious causes such as syphilis, Yersinia, and amebiasis should also be considered. Primary malignancies of the cecum or metastasis from distant sites may present similarly. Other considerations include backwash ileitis from ulcerative colitis, pyogenic abscesses, and fungal infections such as those caused by *Candida* and *Aspergillus*. Each of these conditions can produce symptoms and imaging findings that may be indistinguishable from abdominal TB [6]. The cornerstone of abdominal TB management is anti-TB therapy, with a minimum treatment duration of six months involving a regimen of rifampicin, isoniazid, pyrazinamide, and ethambutol. In some cases, treatment duration may need to be extended. Notably, cure rates of 99% have been reported for six-month treatment regimens [13].

## Conclusions

This case underscores the diagnostic complexity of intra-abdominal TB, particularly in its rare manifestation within the gallbladder, an organ typically resistant to TB infection. The atypical presentation mimicked acute cholecystitis, leading to an unexpected intraoperative discovery that highlights the limitations of conventional diagnostic tools. Despite negative molecular and microbiological tests, histopathological examination played a decisive role in confirming the diagnosis, reaffirming its importance in cases of unexplained intra-abdominal pathology. The necessity of a multidisciplinary approach, integrating surgical intervention with prolonged anti-TB therapy, is crucial for favorable patient outcomes. Given the rarity of gallbladder TB, increased clinical awareness and suspicion are essential to ensure timely diagnosis and management, ultimately improving patient prognosis in similar challenging cases.
